# One-Pot Synthesis of BiCuSO Nanosheets under Ambient Atmosphere as Broadband Spectrum Photocatalyst

**DOI:** 10.3390/nano9040540

**Published:** 2019-04-03

**Authors:** Huanchun Wang, Junping Ding, Haomin Xu, Lina Qiao, Xuanjun Wang, Yuanhua Lin

**Affiliations:** 1 State Key Laboratory of New Ceramics and Fine Processing, School of Materials Science and Engineering, Tsinghua University, Beijing 100084, China; djp15@mails.tsinghua.edu.cn (J.D.); xuhm13@mails.tsinghua.edu.cn (H.X.); qln13@mails.tsinghua.edu.cn (L.Q.); 2 Xi’an Research Inst. of High-Tech, Xi’an 710025, China; wangxj503@sina.com; 3 China Astronaut Research and Training Center, Beijing 100094, China

**Keywords:** BiCuSO, solution route, ambient atmosphere, nanosheets, broadband spectrum

## Abstract

Cuprous based chalcogenides have attracted intensive research interest due to the potential applications in solar energy conversion. However, typical fabrications of these compounds are often carried out under severe conditions, such as inert gas protection, high vacuum, and/or extreme high temperature. Here we reported a one-pot process for cuprous based chalcogenides synthesis in aqueous solution. A strategy for BiCuSO nanosheets fabrication without toxic chemicals or rigorous reagents at pretty low temperatures under an ambient atmosphere was established, with the practicality of morphology controlling and the compatibility of multifarious precursors. Platelike BiCuSO with a thickness range from several to hundreds nanometers are fabricated by adjusting the alkali concentration, reaction time, and temperature. The positive effect of alkali hydroxide concentration is proposed cautiously based on the experimental results. The photocatalytic activities of BiCuSO nanosheet under UV, visible, and near-infrared irradiation were also investigated. BiCuSO obtained at room temperature with a thickness of 4.5 nm showed the most impressive efficiency to decompose organic contaminants. Our research presented a new way for cuprous sulfides fabrication, and might open up a new vista for large-scale synthesis of cuprous based materials as promising broadband spectrum light-absorbing materials.

## 1. Introduction

Compounds with tetragonal ZrCuSiAs type structure were widely studied as promising high temperature superconductors [1]. Beyond that, these materials have interesting properties with respect to thermoelectric performance, transparent semiconducting behavior, or optical applications [2,3,4,5]. However, due to the high melting point of precursors, most of the reported ZrCuSiAs quaternary chalcogenides compounds are synthesized by time-consuming or high temperature process, such as solid state reaction [6,7] and flux technique [8,9,10]. Vacuum or inert atmosphere are usually necessary to preventing cuprous and chalcogenides from oxidation. Analogous processes are extensively applied in chalcopyrite phase ternary or quaternary compounds synthesis [11,12,13]. Moreover, severe condition, such as high temperature and long reaction time, inevitably leads to the growth of crystal along with the difficulty of controlling the morphology of specimens [14]. In the wet-chemical process, the solubility and stability of precursors metal ions in solvent are two important factors that perplex the fabrication of this series of compounds [15,16].

BiCuSO, one of oxysulfides in ZrCuSiAs family, has the same crystal structure and band structure with BiCuSeO and LnCuOS (Ln = La~Lu), the latter two are investigated as promising thermoelectric (TE) materials [17]. Due to the oxidizing of chalcogenides and the cuprous, synthesis of these compounds remains a challenge. Richard synthesized BiCuSO by a two-step solid state reaction in vacuum using Cu_3_BiS_3_ as precursor. [18] The whole process needs longer than 120 h. David Berardan synthesized BiCuSO with stoichiometric mixture of Bi_2_O_3_, Bi_2_S_3_, Bi, Cu, and Cu_2_S sealed in silica tubes under vacuum, and a two-step thermal treatment at 350 °C for 15 h and 600 °C for another one week was needed [19]. Synthesis of these oxychalcogenides through wet chemical approaches is also challenging. Ultra-high pressures or temperature and extremely long reaction time were usually required. For example, Bi_2_O_2_S was synthesized in 10% NaOH solution under 98 MPa pressure for three days at 400 °C [14]. Cu^+^ achieves a maximum molar solubility about 10^−4^ at 200 °C along with the disproportionation of Cu^+^ ions into Cu^2+^ ions and copper metal. To achieve a solubility of 2%~5%, which is a necessary condition to generate a high yield, elevated temperature was employed as the most facilitated method for bismuth compound hydrothermal synthesis [15,16]. Polycrystalline BiCuSO with a size of ∼2 µm was fabricated through hydrothermal process using Bi_2_O_3_, Cu_2_O, and dehydrated Na_2_S as precursors at 250 °C [16]. Similarly, pure BiCuSeO can been synthesized at 250 °C with pressure of 20 atm to promote the dissolution of oxide precursors and stabilize monovalent copper. Hydrazine hydrate was used to prevent the oxidation of dianionic selenium [15]. Among the strategies of cuprous chalcogenides synthesis, air-free, or the inert condition and high temperature are usually employed, which limited its applications. BiCuSO synthesis under mild condition was considered difficult due to the valence state change of Cu in reaction and high melting point of metal sulfide/oxide precursors.

In this work, we present a novel mild wet-chemical method for BiCuSO nanostructures synthesize with convenient morphology control. Water-soluble copper and various bismuth salts are used under ambient atmosphere without high temperature or inert condition. BiCuSO nanosheet with thickness of 4.5 nm is obtained at room temperature. Experiment results indicate that the as-synthesized BiCuSO exhibit efficient photocatalytic activities under ultraviolet, visible, and near infrared light irradiation. Investigations of BiCuSO nanocrystal formation mechanism may contribute to a new understanding of the medium effect in wet chemistry for cuprous based materials fabrication.

## 2. Materials and Methods

### 2.1. Preparation and Characterization of BiCuSO

All chemicals used in the fabrication were of analytical grade and were received without any further purification. In a typical hydrothermal synthesis procedure, 10 mmol Cu(NO_3_)_2_·3H_2_O was dissolved into 60 mL deionized water under magnetic stirring followed with dispersion of 5 mmol Bi_2_O_3_ powder. 40 mmol granulated NaOH was added directly and another 20 min magnetic stirring was needed. After that, 10 mmol thiourea ((NH_3_)_2_CS) was added under vigorous stirring to get uniform mixture. All the operations were carried out under ambient atmosphere. The final mixture was transferred into a 100 mL Teflon-lined stainless steel autoclave and treated at 180 °C for 12 h. After the equipment cooled to room temperature naturally, the black powders were segregated and washed several times with deionized water and absolute ethanol. The final species were dried at 60 °C in oven for further characterizations. BiCuSO prepared with other soluble copper precursors, bismuth oxide precursors were carried out following the same procedure.

For room temperature fabrication, the above-mentioned mixture was stirred incessantly for 24 h in an open baker under ambient atmosphere. Then, the precipitates were washed with deionized water and absolute ethanol, and then dried at 60 °C in an oven.

Powder X-ray diffraction (XRD) was performed on a Bruker D8-Advance diffractometer using monochromatized Cu K_α_ (λ = 0.15418 nm) radiation with a scanning speed of 3°/min. A field emission scanning electron microscope (Zeiss, Merlin Compact, Upper Cohen, Germany) operating at a 10 kV was used to characterize the morphology of the samples. The molar ratio of Bi/Cu for BiCuSO was tested by Atomic absorption spectrometry (AAS, Analytik Jena, Contr AA 700, Jena, Germany). UV-Vis-NIR diffuser reflectance (DRS) measurements were carried out on UV/Vis/NIR spectrometer (PerkinElmer, Lambda 950, Waltham, MA, USA). The Brunauer-Emmett-Teller (BET) surface areas of the samples were analyzed by nitrogen adsorption-desorption measurement using a surface and aperture analyzer (Quantachrome Instruments, Autosorb iQ, Florida, American) after the samples were degassed at 280 °C. The chemical state of elements and bonding characteristics were investigated by X-ray Photoelectron spectroscopy (Thermo Fisher, ESCALAB 250XI, Waltham, MA, USA).

### 2.2. Photocatalytic Activity and Photoelectrochemical Test

The photocatalytic activity of the as-prepared BiCuSO samples was evaluated by photodegrading Congo Red (CR, 100 mg/L) aqueous solution under light irradiation. Typically, 0.16 g photocatalyst was dispersed into 80 mL dye solution and stirred in dark for 2 h in advance to reach the adsorption-desorption equilibrium. Cooling-water bath and magnetic stirring were hold continuously to prevent temperature rise during the degradation process. A 5 W LED with emission wavelength of 365 ± 5 nm was used as the UV light source, and a 300 W xenon lamp with 420 nm and 800 nm cut-off filters was used as visible and NIR light source, respectively. The incident light source was placed above the aqueous solution vertically with an illumination intensity of about 70 mW/cm^2^, 121 mW/cm^2^, and 93 mW/cm^2^ for UV, visible and NIR lights, respectively. At regular time intervals, 3 mL suspension was collected and centrifuged, and the supernatant was analyzed by UV-vis spectrophotometer (UV-3100, Hitachi, Tokyo, Japan).

For photoelectrochemical test, a standard three electrode system on an electrochemical workstation (CHI 660, ChenHua, Shanghai, China) was used. Ag/AgCl and Pt plate were employed as reference electrode and counter electrode respectively in Na_2_SO_4_ solution (0.5 mol. L^−1^). The work electrode was prepared as follow: BiCuSO powder was dispersed in glycol ethylene and stirred for 12 h. Then the slurry was coated onto FTO by doctor blade technology. After being dried at 70 °C for 12 h, the substrate was treated at 250 °C for 2 h in air [20]. The photocurrents of the BiCuSO photoanode under UV, visible and NIR lights irradiation were recorded at a bias of 0 V versus the reference electrode using back irradiation model.

## 3. Results and Discussion

**Figure 1:**
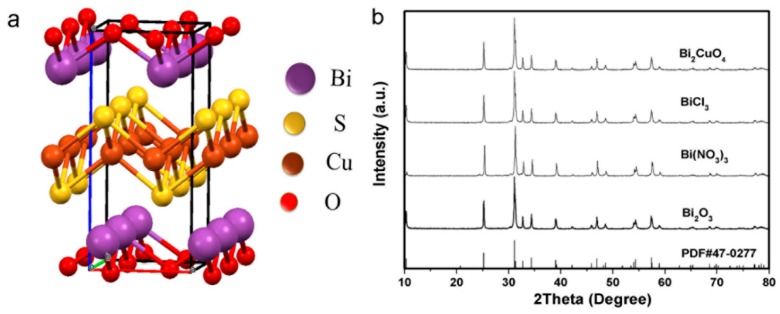
(**a**) Schematic representation of the BiCuSO structure; (**b**) XRD patterns of BiCuSO synthesis by hydrothermal process using various bismuth compounds and Cu(NO_3_)_2_ as precursors in 0.8 mol/L NaOH aqueous, treated at 180 °C for 12 h.

Alkali hydroxide was proposed inapposite for multielements cuprous chalcogenides synthesis [15]. In the present work, the single-phase of samples were obtained conveniently using Bi_2_O_3_ and Cu(NO_3_)_2_ as precursors in NaOH aqueous solution when hydrothermal treated at 180 °C for 12 h. The powder XRD results showed all diffraction peaks could be indexed as BiCuSO according to JCPDS 47-0277 and indicated a tetragonal unit cell (Figure 1a,b). The cationic Bi/Cu ratio of was determined to be 1.00/0.98 by Atomic Absorption Spectrometry (AAS). Control experiments with various NaOH concentration were also carried out. Without NaOH, the mixture was approximately neutral and the final sediments could be indexed as Bi_2_O_2_CO_3_ (Figure S1, Supplementary Materials), scarcely any BiCuSO was detected. Increasing the NaOH concentration to 0.17 mol/L, a small amount of BiCuSO emerged mingling with Bi_2_O_2_CO_3_. It seems that the existent of NaOH contributes to the formation of BiCuSO. Analogous phenomenon was also observed when other bismuth precursors, for example Bi_2_CuO_4_, Bi(NO_3_)_3_, or BiCl_3_, were used (Figure S2, Supplementary Materials). Nevertheless, due to the strong hydrolysis of bismuth compounds such as Bi(NO_3_)_3_ and BiCl_3_ in aqueous, acidic species decreased the pH value and neutralized alkali species, that necessitated the increasing amount of additive alkali hydroxide. Bismuth subcarbonate and wittichenite were major impurities when the alkali hydroxide amount was insufficient. Exceed alkali hydroxide concentration showed no hindering effect on BiCuSO formation according to the experimental result. Entirely pure BiCuSO were obtained in more concentrated aqueous alkali hydroxide (Figure S2a–c, Supplementary Materials). Thiourea are usually employed as the sulfur source in copper chalcogenide compound fabrication [21]. In this work, Cu^2+^ is reduced to Cu*^+^*, which then binds with S released from thiourea. Moreover, Cu*^+^* also bridges the amino groups of thiourea to form complexes [22,23]. The chelation structure promotes the dissolution and stabilization of cuprous during reaction. Employing appropriate temperature and alkali hydroxide concentration in hydrothermal process, CuSO_4_, Cu(NO_3_)_2_, CuCl_2_, and Cu(CH_3_COO)_2_ could be used to fabricated BiCuSO without any extra dehydration treatments or reducing treatments (Figure S2d, Supplementary Materials).

**Figure 2:**
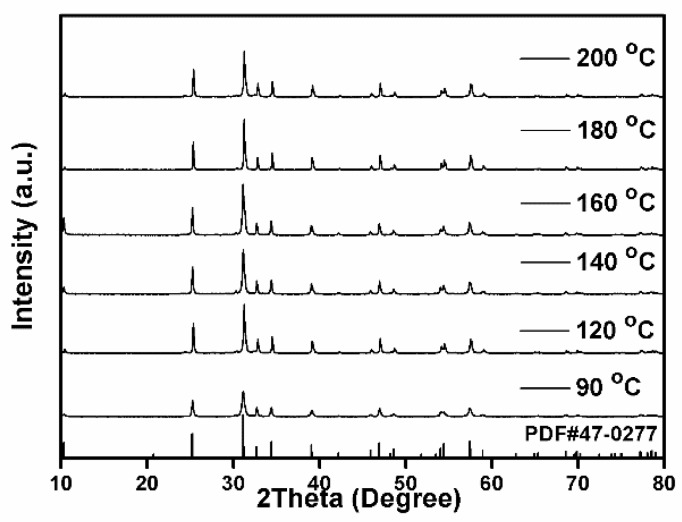
XRD patterns of BiCuSO synthesized by hydrothermal process at different temperatures.

The above experimental results indicate that fabrication of BiCuSO crystal through hydrothermal process was achievable at low temperature. The divalent Cu^2+^ in the precursor was reduced to Cu^+^ in the presence of alkali hydroxide and thiourea. Using Bi_2_O_3_ and Cu(NO_3_)_2_ as metal precursors, fabrication was carried out at different hydrothermal temperatures range from 90 °C to 200 °C for 12 h to get a deeper insight over grow mechanism. As shown in Figure 2, all the specimens were indexed as BiCuSO with ignorable impurities. The crystallization degree, implied by the sharpness of the diffraction peaks, increased when the treat temperatures were higher than 120 °C, which was attributed to the well growth of BiCuSO at higher temperatures.

**Figure 3:**
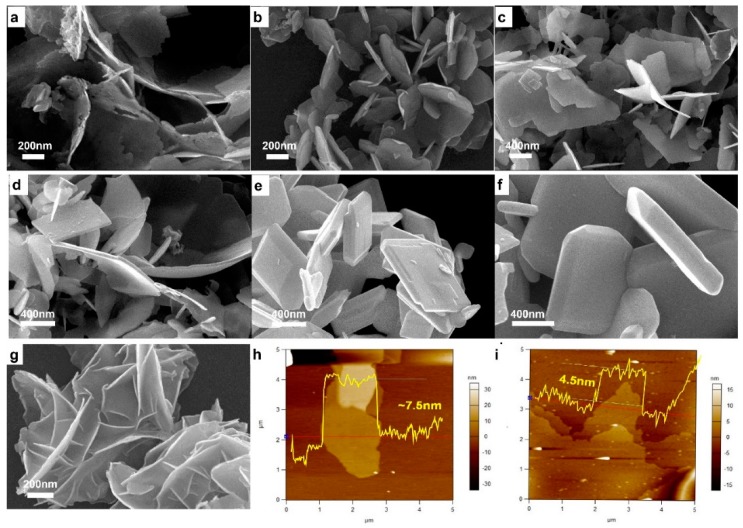
SEM images of BiCuSO fabricated through hydrothermal process for 12 h at various temperature: (**a**) 90 °C; (**b**) 120 °C; (**c**) 140 °C; (**d**) 160 °C; (**e**) 180 °C; and (**f**) 200 °C. (**g**) SEM images of BiCuSO nanosheets fabricated at room temperature by stirring in atmosphere condition. (**h**,**i**) AFM measurements for BiCuSO nanosheets.

Direct morphology evolution was checked by SEM measurements (Figure 3). BiCuSO crystal possessed sheets or plates-like appearances after hydrothermal treatments. The specimen obtained at 90 °C was porous and rough due to the incomplete development of crystal. In range of 120~160 °C, sheets-like BiCuSO were obtained, with the thickness of dozens of nanometers and several hundred nanometers of lateral size. Higher temperatures led to larger sheets-like crystal. When the treating temperatures were higher than 160 °C, polyhedron microcrystalline was obtained. High temperatures promoted the dissolve of tiny crystal and integrity of larger polyhedron, which is deemed as the ripen mechanism. Precipitates dissolve into mother solution and form oversaturated solution during hydrothermal process. Nucleation and re-crystallization occurred and lead to the growth of specific morphologies and the developments of larger crystal. This is the normal strategy for morphology controlling in the hydrothermal process.

Previously, bismuth and its compounds were believed insoluble in alkali hydroxide solution due to strong hydrolysis [24,25], and fabrication of bismuth compounds at low temperature was not easy to realize. In the present work, Bi_2_O_3_ and even the hydrolysis products of other bismuth precursors were easily transferred at extraordinary low temperature. The temperature dependence of the solubility seems inoperative for this strategy. This assumption was further eliminated through fabrication at room temperature (25 °C). Amazingly, without any employment of inert condition or other reductant, BiCuSO was synthesized facilely by stirring the above mentioned mixture under ambient atmosphere for 24 h (using BiCl_3_, Bi(NO_3_)_3_ or Bi_2_O_3_ as bismuth source) (Figure S3, Supplementary Materials). The broaden diffraction peaks of XRD patterns implied much smaller crystal size. Morphology characterization revealed that BiCuSO was irregular hemming nanosheets with thickness less than 10 nm (Figure 3g). Further measurements were preceded using AFM. With several micrometers of in-plane size, BiCuSO nanosheets possessed a thickness thinner than ~4.5 nm (Figure 3h,i). The decrease of geometry size is favor of the increase of surface area. The nitrogen absorption-desorption measurements (Figure S4, Supplementary Materials) revealed that hydrothermal prepared BiCuSO nanocrystals presented an impressive average specific surface area, and lower treat temperature facilitated the increase of specific surface area. The specimen fabricated at room temperature possessed the largest specific surface area (28.166 m^2^/g), that was 2.8 times of that fabricated at 200 °C (9.997 m^2^/g). Thus, a one-pot synthesis strategy of BiCuSO nanosheets under ambient atmosphere at room temperature was developed.

**Figure 4:**
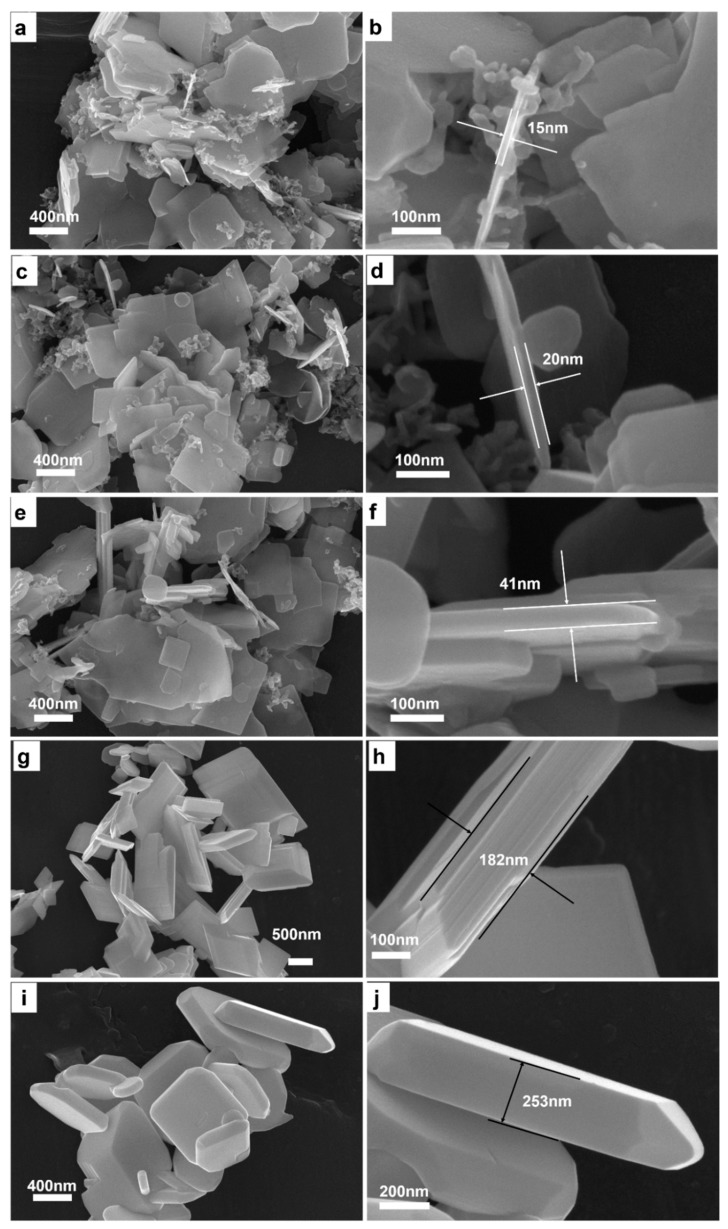
Low magnification SEM images of BiCuSO fabricated through hydrothermal process at 180 °C for various time: (**a**) 0.5 h; (**c**) 1 h; (**e**) 2 h; (**g**) 6 h; and (**i**) 12 h; higher magnification SEM images of BiCuSO fabricated through hydrothermal process at 180 °C for various time: (**b**) 0.5 h; (**d**) 1 h; (**f**) 2 h; (**h**) 6 h; and (**j**) 12 h.

To get deeper understanding of the formation mechanism of BiCuSO crystal in hydrothermal process, the mixtures of precursors were treated at 180 °C for various time respectively. BiCuSO was detected after short time treated at 180 °C. XRD (Figure S5, Supplementary Materials) patterns showed that, after 30 min, the product could be indexed as BiCuSO and bits of impurity was unreacted Bi_2_O_3_. Prolonging the reaction time, impurity disappeared and resulted in pure BiCuSO. SEM images clearly revealed the morphology evolution of BiCuSO at different stages of the hydrothermal process (Figure 4). At the early stage of growth (~30 min), nanosheets with thicknesses of about 15 nm formed. Meanwhile, segmental nanoparticles about dozens of nanometers in diameters coexisted and adjoined to nanosheets. However, the precursor Bi_2_O_3_ is granular and the diameters are about hundreds nanometers (Figure S6a,b, Supplementary Materials). It is suspected that raw precursors transferred to nanosheets and smaller granules at the very beginning of hydrothermal reaction. That indicated BiCuSO sheets grow dynamical easily at more mild condition than reported. Prolonging the reaction time led to the growth of BiCuSO nanosheets, especially the increase of thickness from about 15 nm to 20 nm, 41 nm and 182 nm when terminating the hydrothermal process at 1 h, 2 h, and 6 h. Inerratic polyhedron crystals possessing smooth facets and edges were obtained after 12 h treatment, with the thickness of about 250 nm.

Although the exact chemical mechanism is still unclear, the growth of BiCuSO crystal in this one-pot synthesis strategy can be illustrated as follow: bismuth hydrolysed in NaOH aqueous solution till chemical stabilization was reached in the form of oxy-compound such as BiONO_3_, BiOCl [26,27] or bismuth oxide. Afterwards, with the assistant of NaOH and thiourea, dissolution of bismuth oxy-compound and reduction of bivalent copper ion occured simultaneously, along with the nucleation of BiCuSO nanocrystal at the very beginning when all the raw materials were mixed. The layered crystal structure favored the growth of BiCuSO nanosheets. Nanosheet-like BiCuSO grew up into polyhedron crystal at last.

**Figure 5:**
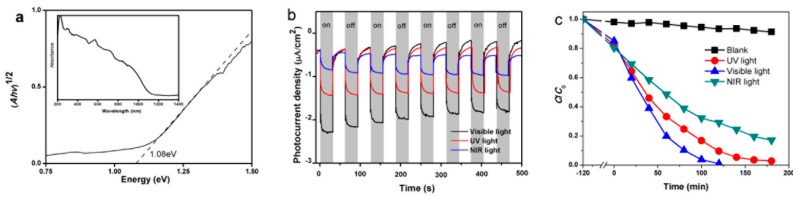
(**a**) UV-visible-Near Infrared diffusion reflection spectrum of BiCuSO, the insert showed the band gap calculated according K-M formula; (**b**) photoelectrochemical (PEC) test of BiCuSO work electrode under the illumination of various light; and (**c**) photocatalytic degradation of CR solution in the presence of BiCuSO nanosheets as photocatalyst with UV, visible, and NIR light irradiation.

Different from previous hydrothermal method, copper salts were universally applicable in this process. Reduction reaction caused by thiourea was responsible for formation of Cu^+^ in BiCuSO. And no extra procedures were needed for the stabilization of Cu^+^ or the inhibition disproportionation of Cu^+^. Due to low-energy of Bi 6*p*, S 3*p*, and O 2*p* states close in energy to the top of the valence band, theoretical investigation showed that the band gap of BiCuSO was ~1.22 eV [28]. Basic properties (dielectric constant, effective mass of photo-generated electron and hole, charge carrier’s mobility) investigated through DFT calculation showed that BiCuSO possesses all the fundamental properties needed for photovoltaic application, and a global efficiency of 10% may be achieved if appropriate solar cells structure was constructed elaborately [28]. As shown in Figure 5, BiCuSO displayed strong absorption capacity in UV, visible and near infrared light according to diffusion reflection spectrum, and experimental band-gap was 1.08 eV (Figure 5a). Samples fabricated at different temperature revealed the same full-spectrum photo-absorption properties (Figure S6c, Supplementary Materials). That means the fabrication condition have no effect on the band gap of different morphology BiCuSO. Photoelectrochemical (PEC) test was performed on electrochemical station under various incident light. BiCuSO demonstrated broad photoconversion capacity, and steep photoelectric responses were got (Figure 5b). Photodegrading of organic dye is usually employed to examine the photocatalytic activity of materials. BiCuSO synthesized at room temperature with the largest specific surface area was investigated. As shown in Figure 5c, the CR solution could be completely degraded in 120 min under visible light irradiation, and around 95.2% of CR was degraded under UV light irradiation in 180 min. Amazingly, BiCuSO nanosheets showed impressive NIR light activity for the photocatalytic degradation of CR, around 78.7% of CR was degraded within 180 min (Figure 5c). The unchanged XRD patterns of BiCuSO nanosheets before and after photocatalysis (Figure S7a, Supplementary Materials) indicated the stability of the photocatalyst. Figure S7b shows the fourier transform infrared (FTIR) spectra of CR powder, BiCuSO nanosheets after adsorption in CR aqueous solution, and that of BiCuSO after completing photocatalytic process. Characteristic peaks of CR powder at 640.33 cm^−1^ and 1178.23 cm^−1^ are identical to that on BiCuSO after adsorption. However, after a whole photocatalytic process, peaks attributed to CR disappeared. The concentration decrease of the CR is not caused by adsorption only. The stability of BiCuSO nanosheets was examined through repeated photooxidation experiments (Figure S7) After four successive cycles, BiCuSO nanosheets degraded around 72% of the CR within 180 min under NIR irradiation and around 91% within 120 min under visible irradiation, which is similar to the activity for the first cycle (78.7% and 98.8 degradation in 120 min). The results again indicated the photocatalytic activity of BiCuSO in broadband spectrum.

**Figure 6:**
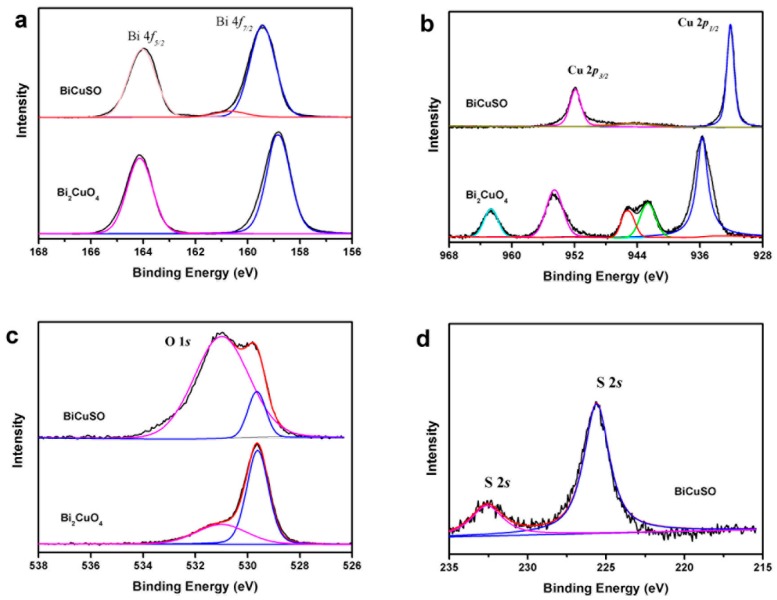
High-resolution XPS scan of (**a**) Bi 4f, (**b**) Cu 2p (**c**) O 1s for BiCuSO nanosheets and hydrothermal prepared Bi_2_CuO_4_, and (**d**) S 2s BiCuSO nanosheets.

**Figure 7:**
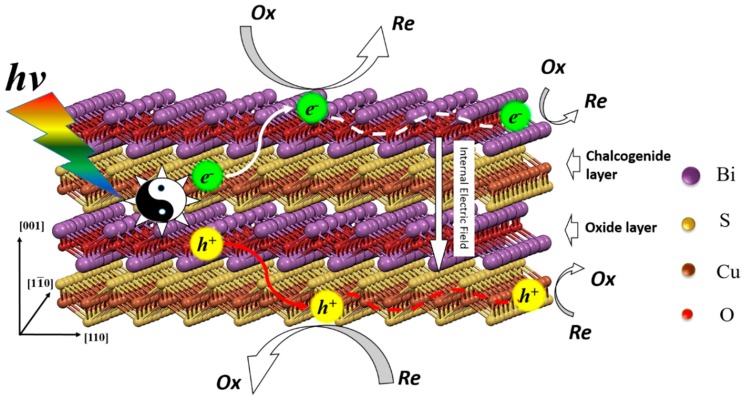
The illustration of photocatalytic process in the layered structure BiCuSO nanosheets.

The effective separations of photo induced holes and electrons is one of the key factors influence the photocatalytic efficiency [29,30]. According to our previous study, the different charge mobility and the effective mass of carriers play important roles in the full spectrum light response ability of BiCuSO nanosheets [20]. However, there was another possibility that the defects can trap holes and electrons and act as reaction sites, which increased the light conversion ability [31,32]. High-resolution XPS was performed to investigate the chemical characteristics of BiCuSO (Figure 6). By comparing the XPS of precursor Bi_2_CuO_4_, which contained Bi^3+^ and Cu^2+^ simultaneously, obvious changes of element chemical states were revealed. Peaks located at 159.11 and 164.22 eV were ascribed to Bi4f _7/2_ and Bi4f _5/2_ in both BiCuSO and Bi_2_CuO_4_. For divalent copper ions in Bi_2_CuO_4_, peaks of Cu2p_3/2_ and Cu2p_1/2_ orbitals located at 954.12 and 934.27 eV were observed respectively, with satellite peaks of higher binding energy. After solution process, monovalent copper characteristics arose with two peaks located at 952.12 and 932.22 eV. Oxygen atom was supposed bonding with bismuth atom in Bi–O layer through hybridization. However, splitting spin-orbit located at 531.17 and 529.38 eV were noticed, and could be ascribed to O2s with △*E*_p_(4f_5/2_ − 4f_7/2_) = 1.79 eV. The splitting feature could be attribute to the interlayer interaction between Bi-O layer and Cu-S layer [33]. That could be furtherly revealed by the peaks located at 232.65 eV which implied the characteristics of sulfate besides the feature of sulfide (peak at 225.7 eV). Overall, the bonding energies of the elements is consistent with the BiCuSO, confirming the formation of pure BiCuSO phase and the reduction of copper through solution process. The XPS results showed that there was no valance change or large amounts of obvious defects in BiCuSO samples. Thus high photocatalytic efficiency was not attributed to these two factors. BiCuOS comprises (Cu_2_S_2_)^2−^ layers alternately stacked with (Bi_2_O_2_)^2+^ along the c axis. Meanwhile, the top of the valence band (VB) is mainly composed of S3*p* and Cu 3*d*, and Bi 6*p* states make major contributions to the bottom of the conduction band (CB). The holes and electrons induced by incident light the irradiation of light were able to migrate in different layers, which was another advantage of the photocatalytic process. The mechanism of the photocatalytic process is illustrated in Figure 7.

## 4. Conclusions

Herein, we reported a providential one-pot strategy for BiCuSO nanostructure fabrication at low temperature under ambient atmosphere, which is free of toxic chemicals or rigorous reagents and is versatile for various bismuth and copper precursors. Alkali hydroxide plays an important part in the synthesis procedure, and a threshold concentration is the prerequisite condition. With the assistant of alkali hydroxide, the reduction of copper ion and the stabilization of cuprous could be easily achieved in-situ delicately. Using the above process, the morphology evolution of BiCuSO crystal during growth reaction was observed. Optimizing the process, BiCuSO nanosheets with thickness of several nanometers can even be obtained under ambient atmosphere at room temperature. We also demonstrated that BiCuSO showed broadband spectrum photo response activity to the range of UV to NIR light. The present work gives the opportunity for large-scale convenient synthesis of cuprous-based semiconductors as promising broadband spectrum light-absorbing materials, and also for morphology construction.

## Supplementary Materials

The following are available online at https://www.mdpi.com/2079-4991/9/4/540/s1, Figure S1: XRD patterns of specimens prepared with various NaOH amount at 180 °C for 12 h; Figure S2: XRD patterns of specimens prepared with various precursors; Figure S3: XRD patterns of BiCuSO prepared at room temperature with various bismuth precursors. Figure S4: nitrogen absorption and desorption curves of BiCuSO nanostructures prepared at differnet hydrothermal temperature; Figure S5: XRD patterns of BiCuSO prepared at 180 °C for various time. Figure S6: SEM images of Bi_2_O_3_; Figure S7: XRD patterns and Fourier transform infrared (FTIR) spectra of BiCuSO before and after photocatalytic process, Cycling runs for the photocatalytic degradation of CR in the presence of BiCuSO under NIR and visible light irradiation.
