# Supplementary material for: One-Pot Synthesis of BiCuSO Nanosheets under Ambient Atmosphere as Broadband Spectrum Photocatalyst
Source: Nanomaterials (Basel). 2019 Apr 3;9(4):540. doi: 10.3390/nano9040540 (PMC6523219; doi:10.3390/nano9040540)
Supplement: Supplementary file 1 [file nanomaterials-09-00540-s001.pdf]

One-Pot Synthesis of BiCuSO Nanosheets under Ambient Atmosphere as Broadband Spectrum Photocatalyst

Huanchun Wang [1,2,*], Junping Ding [1,3], Haomin Xu [1], Lina Qiao [1], Xuanjun Wang [2], and Yuanhua Lin [1,*]

[1] State Key Laboratory of New Ceramics and Fine Processing, School of Materials Science and Engineering, Tsinghua University, Beijing 100084, People's Republic of China; wang-hc12@tsinghua.org.cn (H.W.); djp15@mails.tsinghua.edu.cn (J.D.); xuhm13@mails.tsinghua.edu.cn (H.M.); qln13@mails.tsinghua.edu.cn (L.Q.); linyh@mail.tsinghua.edu.cn (Y.L.)

[2] Xi'an Research Inst. of High-Tech, Xi'an, Shaanxi Province, 710025, China; wangxj503@sina.com (X.W.)

[3] China Astronaut Research and Training Center, Beijing 100094, People's Republic of China;

* Correspondence: wang-hc12@tsinghua.org.cn (H.W.); linyh@mail.tsinghua.edu.cn (Y.L.); Tel.: +86-29-84744342(H.W.)

Keywords: BiCuSO, solution route, ambient atmosphere, nanosheets, broadband spectrum.

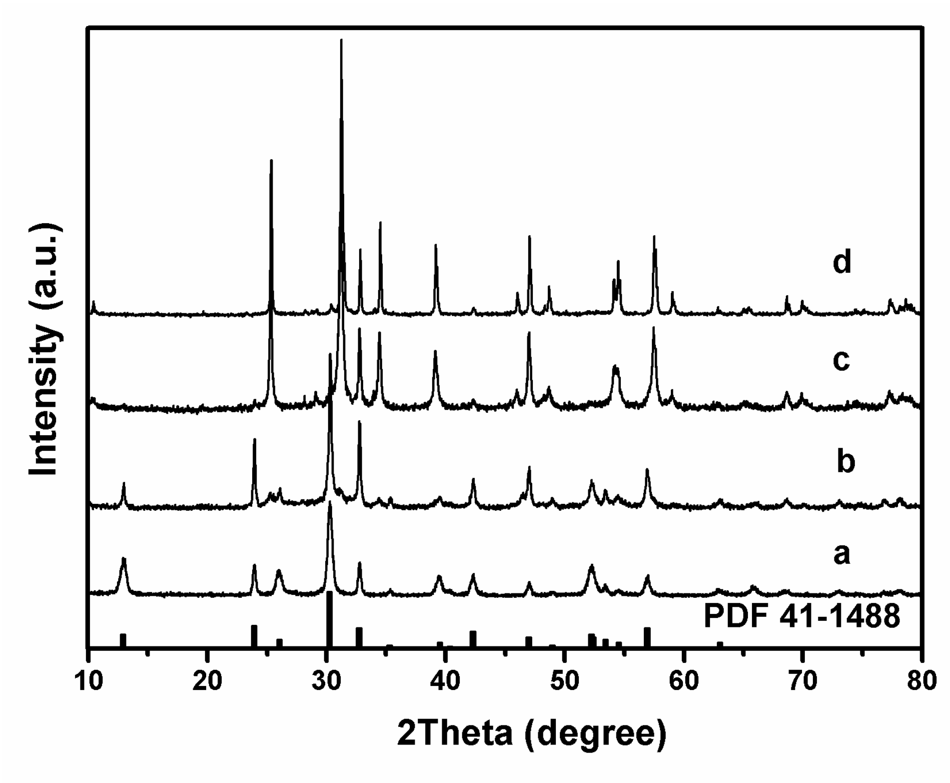

Figure 1. XRD patterns of specimens prepared with various NaOH amount at 180°C for 12h (a) without addition of NaOH; (b) 0.17mol/L NaOH; (c) 0.67mol/L NaOH; and (d) 2 mol/L NaOH. The bottom shows the PDF standard card of $Bi_2O_2CO_3$.

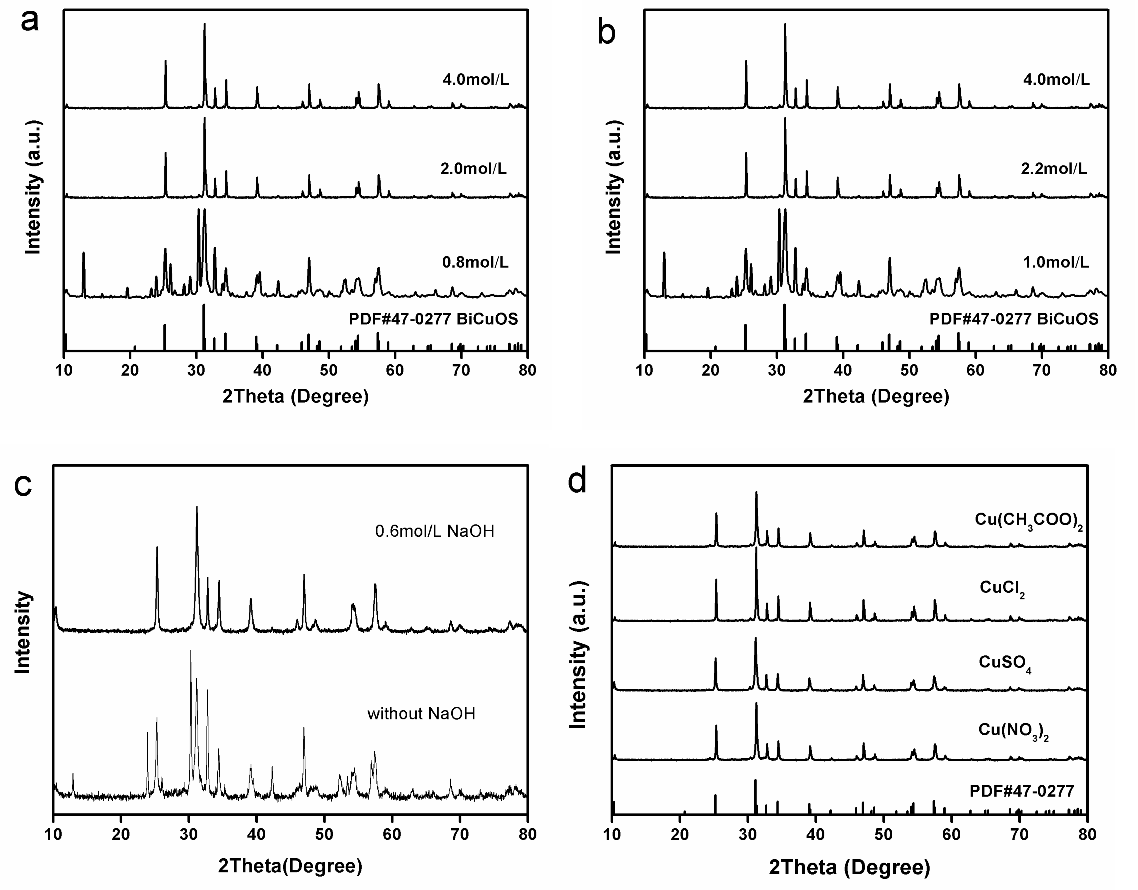

Figure S2 XRD patterns of specimens prepared at various precursors under different condition. Preparation in different concentration NaOH aqueous solution with various bismuth precursors: (a) $Bi(NO_3)_3$; (b) $BiCl_3$; (c) Bi_2CuO_4. $CuNO_3$ was used as copper precursor uniformly. (d) XRD patterns of specimens prepared using various copper salts as precursors with Bi_2O_3 as bismuth precursors. All the preparation were carried out at 180°C for 12h.

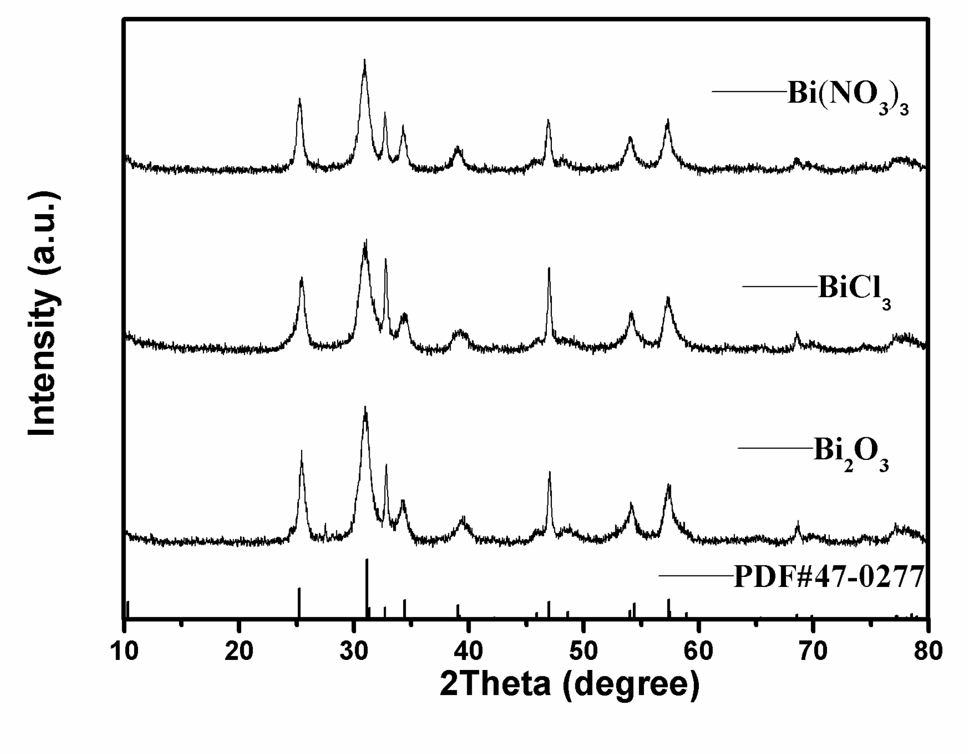

Figure S3 XRD patterns of BiCuSO prepared at room temperature with various bismuth precursors. All the specimens were fabricated through stirring for 24h.

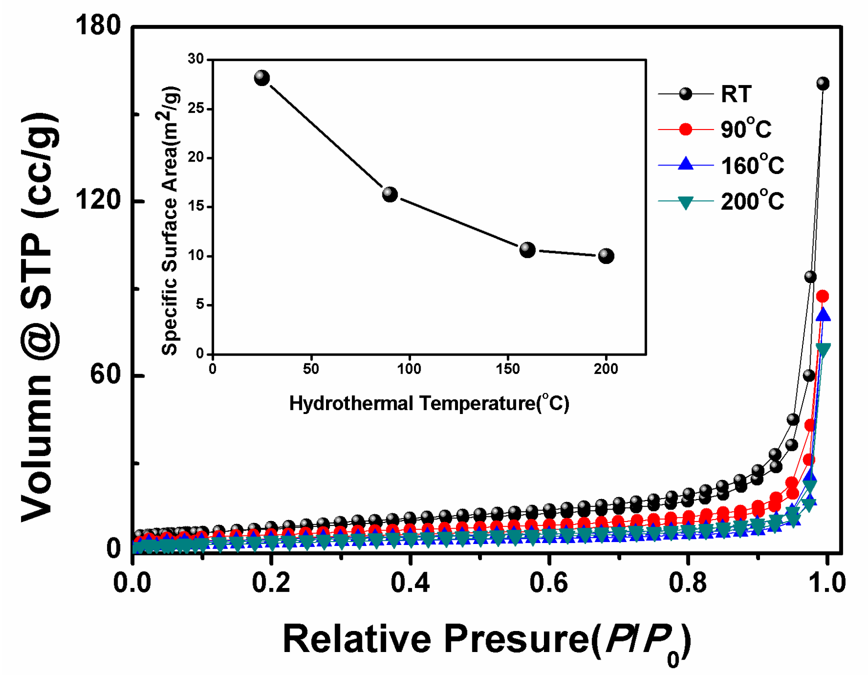

Figure S4 nitrogen absorption and desorption curves of BiCuSO nanostructures prepared through hydrothermal process at 90°C, 160°C, 200°C and through room temperature process. The insert shows the BET surface area of BiCuSO prepared at different treat temperature.

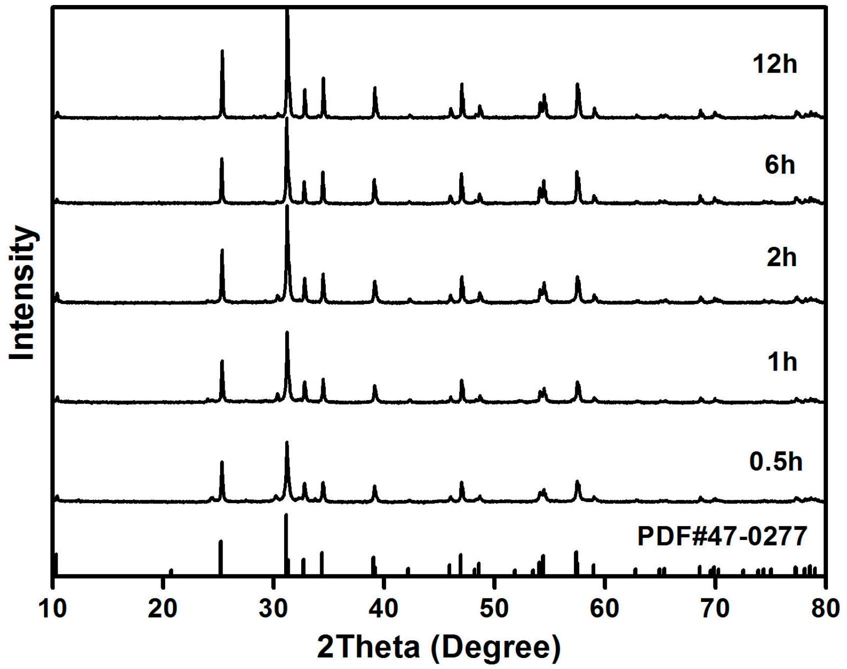

Figure S5 XRD patterns of BiCuSO prepared at 180°C for various time. All the specimens could be indexed as BiCuSO according to PDF card.

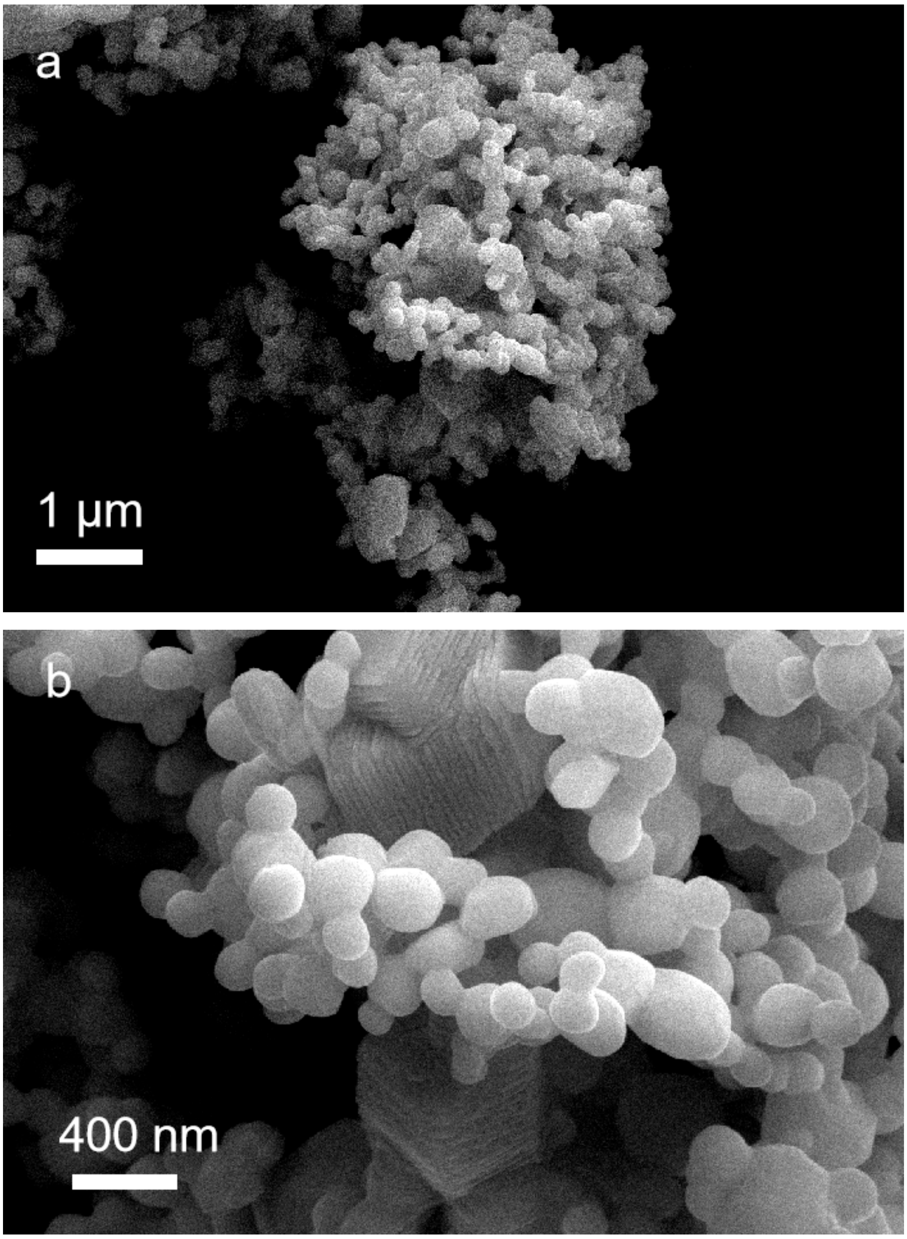
a
1 μm
b
400 nm

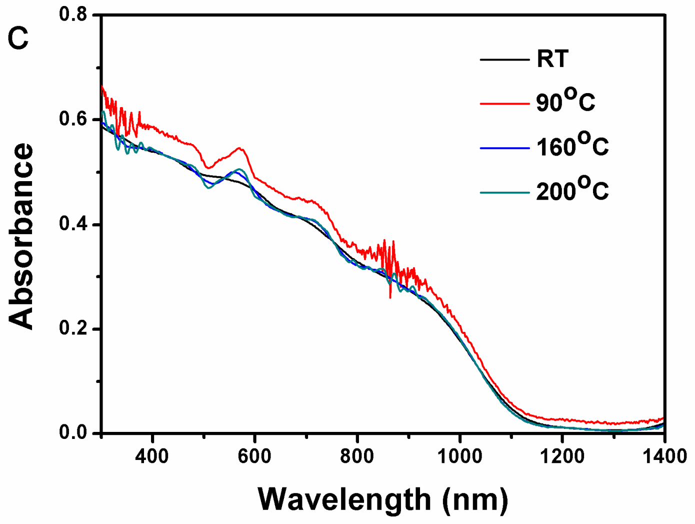

Figure S6 (a,b)SEM images of Bi_2O_3 which is used as precursor of bismuth without any further treatments. Bi_2O_3 possess anomalous granular morphology with diameter of about 200nm.(c)UV-visible-Near Infrared diffusion reflection spectrum of BiCuSO nanostructures prepared at 90°C, 160°C, 200°C and room temperature

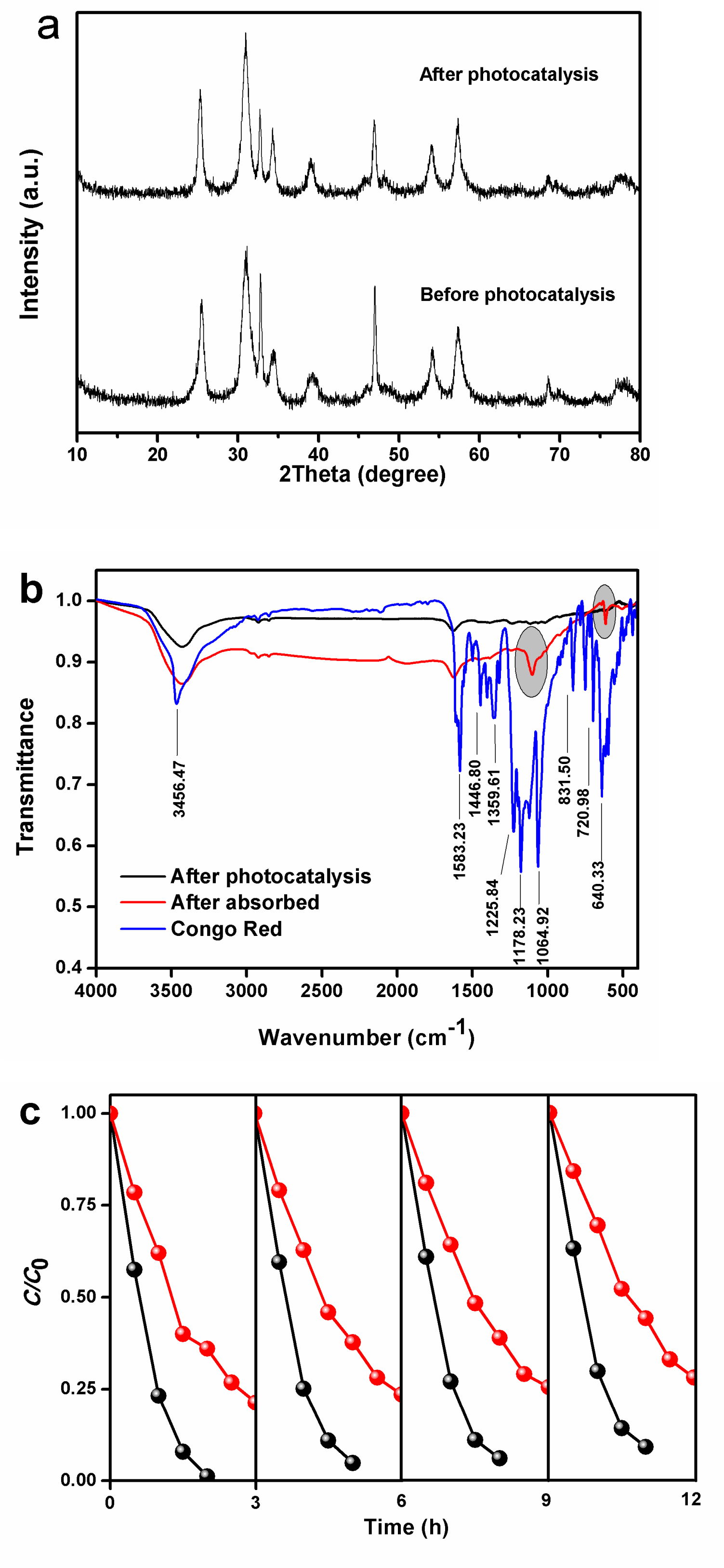

Figure S7 (a) XRD patterns of BiCuSO before and after photocatalytic process. (b)

Fourier transform infrared (FTIR) spectra of the CR powder, BiCuSO nanosheets after adsorbed in CR aqueous solution for 30 min, as well as BiCuSO nanosheets after fully photocatalytic degradation.（c）Cycling runs for the photocatalytic degradation of CR in the presence of BiCuSO under NIR and visible light irradiation.
